# Supplementary material for: The association between Toxoplasma gondii infection and asthma in the United States: A cross-sectional survey analysis
Source: PLoS One. 2025 Jul 28;20(7):e0304044. doi: 10.1371/journal.pone.0304044 (PMC12303259; doi:10.1371/journal.pone.0304044)
Supplement: Attachment — Submitted filename: Response_to_reviewers_Anholt_2025April29.docx [file pone.0304044.s002.docx]

Dear Editors and Reviewers,

Thank you for your thoughtful comments on the manuscript, *“The association between Toxoplasma gondii infection and asthma in the United States: A cross-sectional survey analysis.”* I especially appreciate your suggestions regarding improvements to the structure and narrative of the abstract and introduction, which are incorporated into the revised version. The manuscript has also undergone further review by a Wiley associate editor.

Please accept my sincere apologies for the delay in this resubmission. I was unaware that the initial review had been completed, and unfortunately, after following up with the PLOS ONE editorial office, I experienced a prolonged illness due to pneumonia, which further delayed my response.

All requested edits and revisions to the manuscript are outlined below. Please note that I have changed my contact email to hezyvet@gmail.com, because of issues receiving emails from PLOS One at my University of British Columbia student email address. Thank you again for your time and valuable feedback.

Sincerely,
Heather Anholt

**Record of changes to the manuscript**

- I have changed the level 1 headings to sentence case.
- I have renamed the figure tiff files.
- I have changed the citation style from Vancouver to PLOS ONE.
- I have made tracked formatting changed to the manuscript in accordance with:

<https://journals.plos.org/plosone/s/file?id=wjVg/PLOSOne_formatting_sample_main_body.pdf>

and

<https://journals.plos.org/plosone/s/file?id=ba62/PLOSOne_formatting_sample_title_authors_affiliations.pdf>

**Reviewer 2: Clarity of Reporting**
Abstract: The abstract of the study would benefit from a more comprehensive reference to the principal findings. Emphasizing these pivotal conclusions in the abstract will furnish readers with a more lucid comprehension of the study's importance and findings from the beginning. Highlighting the principal findings in this part will augment the abstract’s efficacy in communicating the study’s significance and relevance.

**lines 22-30: The following text has been added to the Abstract.**

Of the 12,620 subjects tested for *T. gondii* infection, 89.2% were seronegative and 10.8% seropositive. No asthma was reported by 83.5% of subjects, while 16.5% reported varying degrees of asthma severity. We detected no significant association between *T. gondii* infection and asthma. While the unadjusted regression model suggested a small protective effect of *T. gondii* on asthma (OR = 0.90; 95% CI = 0.83-0.97), no effect was detected when the model was adjusted for key demographic factors (OR = 1.00, 95% CI = 0.91-1.10). While *T. gondii* may be a marker for the protective effect of exposure to a diversity of microbial organisms early in life, it has no apparent causal effect on asthma, or it may not be significant when considered in isolation.

**Reviewer 2: Introduction**

It is advised that one paragraph be devoted to exploring the influence that T. gondii has on the immune system in the introduction. Focus should be placed on the important components that contribute to the emergence of an allergic response.

**lines 48-63: the last paragraph of the Introduction now reads as follows:**

*Toxoplasma gondii* is an obligate intracellular gastro-intestinal protozoan, and nearly ubiquitous microorganism, whose definitive host is cats. People become infected with *T. gondii* through indirect fecal-oral transmission or consumption of undercooked meat. About 12% of the U.S. population is infected, and in some countries, prevalence is as high as 60% [7,8]. Infection with *T. gondii* induces a strong cell-mediated immune response, with the production of IFN-g cytokines by polarized T helper type 1 cells (Th1) in the early stages of infection [9,10]. Conversely, the pathophysiology of asthma is characterized by the development of hyperreactivity due to cytokines produced by T helper type 2 cells (Th2). Infection with *T. gondii* may exert a protective effect on the development of antigen-induced airway inflammation through high concentrations of Th1 cytokines and a reduction of allergen-specific Th2-associated cytokines associated with the *T. gondii* immune response [11]. Indeed, Fenoy *et al.* (2009) showed that *T. gondii* infection substantively blocked the development of antigen-induced airway inflammation in adult laboratory (BALB/c) mice [9]. Despite recognized impacts on the mammalian immune response, however, the relationship between *T. gondii* infection and asthma has not been well investigated [6,8]. In the current study, we quantify the association between *T. gondii* infection and asthma in the U.S. population.

**Reviewer 2: It is strongly recommended that the article undergo a thorough review for grammar and writing.**

- The manuscript has been reviewed by a Wiley associate editor and the following changed have been made in response to their comments.
- Tables 2 and 3 appeared in reverse order. They have been renumbered.
- The following text has been added to **lines 169-170 (Table 2):**
  - “AIC does not offer a comparison of model fit across age categories because they are not identical data sets.”
- **lines 231-233:**
  - This paragraph has been edited to avoid direct comparison of AIC values across data sets. It now reads:

Regression analyses stratified by age category suggested that there is some effect modification by age; the association between *T. gondii* infection and asthma severity varied across age categories, but within each category the association remained insignificant (Table 2).

- **lines 235-237**
  - the main result is reiterated in the first sentence of the Discussion as follows:

No significant relationship was detected between *T. gondii* infection and asthma in U.S. residents from the 2012-2014 NHANES survey cycles when models were adjusted for key demographic factors.

- **line 253:** “might” has been changed to “would likely”.
